# DNA and RNA Extraction and Quantitative Real-Time PCR-Based Assays for Biogas Biocenoses in an Interlaboratory Comparison

**DOI:** 10.3390/bioengineering3010007

**Published:** 2016-01-13

**Authors:** Michael Lebuhn, Jaqueline Derenkó, Antje Rademacher, Susanne Helbig, Bernhard Munk, Alexander Pechtl, Yvonne Stolze, Steffen Prowe, Wolfgang H. Schwarz, Andreas Schlüter, Wolfgang Liebl, Michael Klocke

**Affiliations:** 1 Bavarian State Research Center for Agriculture, Department for Quality Assurance and Analytics, Lange Point 6, 85354 Freising, Germany; Bernhard.Munk@lfl.bayern.de; 2 Leibniz Institute for Agricultural Engineering Potsdam-Bornim, Department Bioengineering, Max-Eyth-Allee 100, 14469 Potsdam, Germany; jderenko@atb-potsdam.de (J.D.); arademacher@atb-potsdam.de (A.R.); mklocke@atb-potsdam.de (M.K.); 3 Beuth University of Applied Sciences, Department of Life Sciences and Technology, Luxemburger Strasse 10, 13353 Berlin, Germany; shelbig@beuth-hochschule.de (S.H.); steffen.prowe@beuth-hochschule.de (S.P.); 4 Department of Microbiology, Technische Universität München, Emil-Ramann-Str. 4, D-85354 Freising-Weihenstephan, Germany; alexander.pechtl@tum.de (A.P.); wschwarz@wzw.tum.de (W.H.S.); wliebl@mytum.de (W.L.); 5 Institute for Genome Research and Systems Biology, CeBiTec, Bielefeld University, Bielefeld, Germany; ystolze@cebitec.uni-bielefeld.de (Y.S.); aschluet@cebitec.uni-bielefeld.de (A.S.)

**Keywords:** ring-trial, fermenter sludge, DNA purity, PCR suitability, extraction efficiency, RNA preservation, reverse transcription, methanogens, *Bacteria*, absolute quantification

## Abstract

Five institutional partners participated in an interlaboratory comparison of nucleic acid extraction, RNA preservation and quantitative Real-Time PCR (qPCR) based assays for biogas biocenoses derived from different grass silage digesting laboratory and pilot scale fermenters. A kit format DNA extraction system based on physical and chemical lysis with excellent extraction efficiency yielded highly reproducible results among the partners and clearly outperformed a traditional CTAB/chloroform/isoamylalcohol based method. Analytical purpose, sample texture, consistency and upstream pretreatment steps determine the modifications that should be applied to achieve maximum efficiency in the trade-off between extract purity and nucleic acid recovery rate. RNA extraction was much more variable, and the destination of the extract determines the method to be used. RNA stabilization with quaternary ammonium salts was an as satisfactory approach as flash freezing in liquid N_2_. Due to co-eluted impurities, spectrophotometry proved to be of limited value for nucleic acid qualification and quantification in extracts obtained with the kit, and picoGreen^®^ based quantification was more trustworthy. Absorbance at 230 nm can be extremely high in the presence of certain chaotropic guanidine salts, but guanidinium isothiocyanate does not affect (q)PCR. Absolute quantification by qPCR requires application of a reliable internal standard for which correct PCR efficiency and Y-intercept values are important and must be reported.

## 1. Introduction

Since its invention in 1983, polymerase chain reaction (PCR) and PCR based techniques for analyzing DNA started a triumphal march in many segments of life science research and biotechnological applications, and represented a break-through in culture independent analysis of biological specimens from diverse ecosystems. The same was true for the quantitative PCR variant, the Real-Time quantitative PCR (qPCR) [1], which was introduced in the mid-1990s. Several technically different qPCR variations were developed [2], of which systems using a fluorescent DNA binding dye or a hydrolysis probe in 5′-nuclease assays are most widely applied. By integration of a reverse-transcription (RT) step after digestion of genomic DNA, it is intended to quantify RNA species by RT-qPCR.

Providing quantitative data in the analysis of biogas biocenoses is a major task not only for research but particularly for practice. For example, quantitative assessment of functionally important microbial guilds and their activity can be used for process diagnosis and control. As recently shown, novel early warning systems of biogas process failure based on qPCR and RT-qPCR analyses announced microbial metabolic stress weeks before the response of conventional chemical parameters [3,4]. However, the quality of (RT-)qPCR analyses is directly dependent on the quality of the nucleic acid extract which depends on the applied sample processing measures and the extraction procedure. Extract quality thus predetermines suitability for the intended analysis. Some steps of these sample processing and analysis pipelines entail uncertainty. This is expressed, e.g., in the enormous number on “the best” DNA or RNA extraction routine for PCR based approaches for challenging samples such as from biogas fermenter sludges or similar matrices, e.g., [5,6,7,8]. However, the suggestions made are partly contradictory. These challenges and uncertainties are more detailed in the following:

If the concentration of certain organisms, genes or transcripts is to be specifically determined in environmental samples or other specimens, absolute quantification of nucleic acid copies by qPCR or RT-qPCR currently is the method of choice. Since qPCR and, particularly, RT-qPCR approaches present some pitfalls, details of crucial analysis steps and data of control points should be reported [9,10]. This is particularly important if it is intended to compare the results with those of other laboratories. The efficiency of the qPCR reaction can be calculated and should not be below 90% and above 110% [11]. Optimum PCR efficiency is usually obtained if the concentration of inhibitors in the extract is minimized (see below). PCR inhibition can be detected in a dilution assay, allowing the elimination of inhibited reactions from further evaluations.

Processivity, robustness and reaction environment of DNA-polymerases have greatly been improved since their first introduction. Much less is known about the efficiency of RT reactions with RNA extracts. Different RT enzymes and chemistries are available on the market but information on RT inhibitors and modes of RT inhibition and respective research is scarce, although the RT reaction is located upstream of PCR and is thus more prone to co-extracted inhibitors. Absolute quantification of RNA by RT-qPCR is thus a prominent challenge for the future.

Reliable nucleic acid quantification requires not only efficient PCR- (and RT-) reactions, meaning that the concentration of inhibitory compounds in the DNA and RNA extracts must have been sufficiently removed. The extraction routine should also provide high extraction efficiency conferring high assay sensitivity. Moreover, different nucleic acid isolation procedures for biogas biocenoses from fermenter samples can considerably impact the DNA composition in the extract [6,7], e.g., by selective lysis of different microorganisms. This can result in biased detection of taxonomic groups. Nucleic acid extraction is thus a tradeoff between sample purity on the one hand and maximum recovery rate on the other hand.

Extraction of nucleic acids has a longer history than PCR applications. Phenol/chloroform-based extraction techniques were mainly developed to yield nucleic acids as pure as possible, e.g., for DNA/DNA hybridization studies. Nucleic acid purity is conventionally judged by spectro-photometrical absorption at 260 nm, 280 nm and 230 nm and benchmark thresholds or intervals for the corresponding ratios A260/280 and A260/230 [12]. However, several drawbacks are associated with phenol/chloroform-based extraction techniques such as working with hazardous chemicals in sometimes many repetitive steps resulting often in phenol contamination of the extract and (partial) inhibition of subsequent PCR, as well as poor extraction efficiency (see below), precluding work with small sample volumes. Moreover, recent findings challenge the above mentioned thresholds for nucleic acid purity if extracts are intended to be used for PCR approaches. For example, Munk *et al.* [13] reported very low A260/230 ratios in DNA extracts, probably due to the presence of guanidine (iso)thiocyanate (GITC) which is used in several more user-friendly nucleic acid extraction kits. GITC absorbs strongly at 230 nm, but no inhibition of qPCR reactions was observed in spite of its presence. Since such kits are becoming more and more popular, their suitability and efficiency for (RT-)qPCR-based approaches had to be evaluated in the present work. Kit-based systems, however, mostly use small sample volumes, resulting in relatively low nucleic acid yields. This can be limiting for downstream applications requiring higher nucleic acid amounts. Moreover, harsh sample disruption can result in too strong nucleic acid fragmentation for analyses requiring longer template strands. In the case of total RNA extraction, this can result in compromised RNA quality as indicated by low RIN (RNA integrity number) values [14], impeding e.g., metatranscriptome analysis, as is addressed in this paper. The destination of the extract must therefore determine the extraction methodology used.

A further problem associated with RNA and, particularly, mRNA is its instability. If samples are taken from biologically active environments such as biogas fermenters, RNA degradation must be obviated at once to avoid biased results. They should thus either immediately be processed or treated by physical or chemical means preventing RNA decomposition. Several approaches of RNA preservation have been developed with snap-freezing in liquid nitrogen as an effective and widespread method. More recently, quaternary ammonium salts have been reported to be efficient RNA preservatives in some [15,16] but not all [17] studies. It should be evaluated in the present study, which RNA preservation method may be suitable for samples from biogas fermenter sludge.

For absolute quantification by (RT-)qPCR, the extraction efficiency, the DNA or RNA recovery rate, must be known to account for losses during the extraction procedure. The recovery rate can be determined, e.g., by sample spiking with pre-quantified standards [18]. Without these data, absolute concentrations of target genes or transcripts cannot be calculated reliably in environmental samples. However, respective information is only rarely presented in studies reporting quantitative results, and, particularly, data on RNA extraction efficiencies are almost inexistent. This paper will show an example of how DNA extraction efficiencies can be assessed.

The generation of a reliable standard is a further prerequisite for absolute quantification, and the few papers dealing with this subject indicate that this is obviously a neglected issue. It is strongly suggested to apply an internal standard. Dilution series of the standard should result in a standard curve with qPCR efficiency close to 100% (slope close to −3.3), indicating absence of PCR inhibitors, and with most qPCR platforms, a Y-intercept between Cq (cycle of quantification) values of about 34–39 [19]. Lower Y-intercept values of the standard will result in considerable underestimation and higher values in substantial overestimation of the target in the sample.

With this background and considering the challenges associated with nucleic acid extraction from difficult matrices such as biogas digester sludge and subsequent (RT-)qPCR analysis, this communication will address the following questions and issues:

- Does a given kit-based DNA extraction protocol (with small modifications) yield similar qPCR results in a ring-trial in an interlaboratory comparison? Do modifications that were introduced by some partners impact the results, and which differences can be seen if a traditional chloroform-based routine is used?
- How efficient is the extraction considering that maximum assay sensitivity, *i.e.*, lowest possible method detection limit should be provided?
- How suitable are the extracts for qPCR? If PCR inhibition is observed, which qualities do the extracts have?
- Do the different qPCR protocols that were applied in the ring-trial yield different results? Are there any peculiarities explaining the differences?
- Which are the most important process steps, control points and benchmarks that should be respected in the qPCR analysis pipeline, and are traditional absorption ratio thresholds helpful?
- Which methods of RNA preservation can be recommended for biogas fermenter sludge samples?
- Which pitfalls do the different RNA extraction protocols present for different purposes such as RT-qPCR and metatranscriptome analysis?

## 2. Experimental Section

### 2.1. General Outline of the Experiments

Five partners (A–E) participated in the interlaboratory comparison of nucleic acid extraction and RNA preservation methods.

Partner A operated four two-stage two-phase biogas fermenter systems and partner B four single-stage biogas fermenters digesting grass silage, as specified below. All biogas systems were fed with grass silage originating from the same stock. The silage was provided as big-packs by partner B.

In an initial analysis, partner B compared different DNA extraction kits in a standard-spiking approach which was described to be suitable for the given plant-based fermenter sludge matrix. A kit-based routine was optimized yielding a DNA recovery rate of about 90%. In the following DNA extraction ring-trial, all five partners participated. Four of these (partners A–D) served as analysts performing quantitative Real-Time PCR (qPCR) with each of the DNA extracts provided by the five partners. The analysts used their established in-house routines (see Section 2.7) targeting *Bacteria* (protocols i, iv and v), *Archaea* (protocol ii) or methanogenic *Archaea* (protocol iii).

In order to compare RNA preservation methods, digestate from the “hydrolysis” stage of partner A was treated by four different preservation methods as specified below and sent to partner B for RNA extraction, reverse transcription and cDNA synthesis following two different protocols (see below). cDNA was quantified for methanogenic *Archaea* directly by partner B (protocol iii) and after shipping cDNA to partner A for *Bacteria* (protocol i) and *Archaea* (protocol ii).

**Figure 1:**
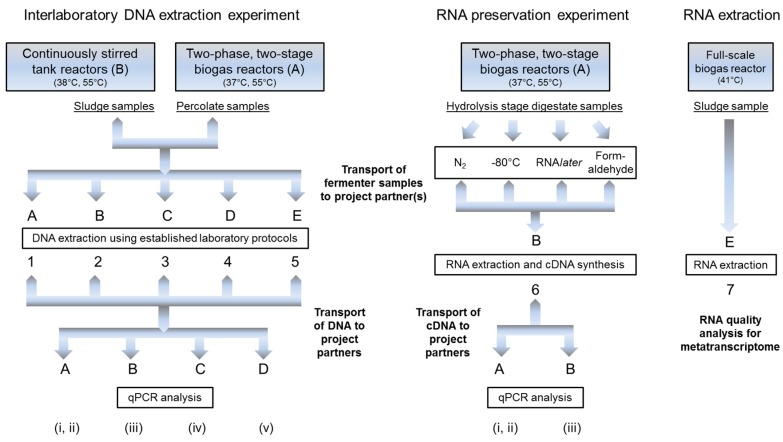
Experimental flow chart of the interlaboratory DNA extraction and RNA preservation experiments; numbers refer to laboratory protocols, letters to partners.

Flow charts of the experimental setup of the interlaboratory DNA extraction and the RNA preservation experiment are shown in Figure 1.

### 2.2. Substrate, Reactor Set-Up and Sampling

Grass silage was provided by partner B in big-packs. It was fed to the single-stage fermenters operated by partner B (see below) and shipped to partner A as substrate for its two-stage two-phase fermenter systems. The grass silage showed the following properties: organic dry matter (ODM) = 32.57% fresh matter (FM); NH_4_^+^-N = 0.63 g·kg_FM_^−1^; pH = 5.11; volatile organic acids (VOA) = 8.26 g·kg_FM_^−1^. There was no hint for spoilage or minor quality. Partner A chopped the silage to approximately 3 cm, froze aliquots and fed them after defrosting in batches to the “hydrolysis” stages. Partner B chopped the silage to about 1 cm after freezing, prepared daily portions and fed aliquots to the single-stage fermenters.

Partner A operated two mesophilic (37 °C) and two thermophilic (55 °C) two-stage two-phase biogas fermenter systems. The principle of the reactor set up was described in detail by Schönberg and Linke [20]. Each system consisted of one hydrolysis reactor (HR), one percolate (process fluid) storage tank and one anaerobic filter compartment. The HR reactors were fed with grass silage (see above) in batches every 28 days. After one batch, the digested substrate (digestate) was replaced by fresh grass silage. The subsequent percolate storage tank supplied process fluid for the semi-continuously percolated HRs and the anaerobic filter compartment which contained biofilm carriers. The substrate load of the HRs was successively increased from 250 g to 500 g FM, which was applied in a start-up procedure of five batch fermentations to ensure adaptation of the microbial community. Digestate was taken from the HRs after four batches (112 days) of the start-up procedure at high methane yield (356.5 ± 8.5 L_STP_·kg_VS_^−1^). It was treated instantly with four different preservation methods to evaluate the most efficient one for RNA stability (Figure 1). Percolate samples were taken from the HRs and immediately frozen for interlaboratory comparison of DNA extraction and qPCR analysis (Figure 1) after five batch runs (140 days) at high methane yield (370.2 ± 4.2 L_STP_·kg_VS_^−1^).

Partner B operated two mesophilic (38 °C) and two thermophilic (55 °C) continuously stirred tank reactors (CSTRs) with the grass silage in parallel. At the time of sampling, the organic loading rate was relatively low (0.5 g_VS_·L^−1^·d^−1^), and the process ran stably and very efficiently with a methane yield of about 400 L_STP_·kg_VS_^−1^ at both temperatures. The concentration of short-chain fatty acids (SCFA) was low, all SCFA were below 500 mg·L^−1^. Free ammonia-nitrogen (FAN), however, was continuously rising, and amounted to about 500 mg·L^−1^ and 1500 mg·L^−1^ in the meso- and thermophilic processes, respectively. Although there were no symptoms of process disturbance yet, the rising FAN and, particularly, the already high FAN values in the thermophilic process suggested precaution in the further operation strategy [3].

### 2.3. Comparison of DNA Extraction Kits and DNA Recovery Rates

It has been shown in a previous study [5] that initial repeated washing of cattle manure and samples from fermenters operated with renewable resources greatly reduced co-elution of PCR-inhibitors in DNA extraction. The washing procedure was consequently integrated in DNA and RNA extraction protocols [13,21]. With this initial modification, different DNA extraction kits (FastDNA™ SPIN Kit for Soil, MP Biomedicals (FSKS); UltraClean^®^ Soil DNA Isolation Kit, MoBio Laboratories Inc. (USIK); QIAamp DNA Stool Mini Kit, QIAGEN (QSMK)) were compared. The comparison was based on a standard spiking approach:

A pre-quantified *Escherichia coli* suspension (10 μL) was spiked to 390 μL of an autoclaved cattle manure sample. The absence of *E. coli* DNA in the sample was confirmed using the DNA extraction protocol of Lebuhn *et al.* [5] and the qPCR protocol given below. After mixing thoroughly and washing with PBS and 0.85% KCl (centrifugation steps 5 min at ca. 12,000 g), DNA was extracted from 40 μL sample aliquots in parallel with the three DNA extraction kits in accordance with the manufacturers’ recipes. 5′-nuclease qPCR assays specific for *E. coli* were carried out for undiluted extracts and serial (1:10) extract dilutions in triplicate in 25 μL reaction volumes containing 0.6 μM primers Ec_mur_27f (GATAAATTTCGTGTTCAGGGGCCA) and Ec_mur_177r (CTTTCGCACCCAGCTGGCTT), 0.2 μM hydrolysis probe Ec_mur_126S (5′-FAM-AGAACCGGTAGAGATCCAGAACGTCC-TAMRA-3′), 0.75 U Platinum *Taq* polymerase (Invitrogen) and 1 x supplied PCR buffer (without MgCl_2_), 200 μM dNTPs, 6 mM MgCl_2_ and 2.5 μL DNA template. The relatively high MgCl_2_ concentration was generally used by partner and analyst B because it was shown in a previous experiment with a DNA extract from cattle manure spiked with *E. coli* that 6 mM MgCl_2_ in the given qPCR assays provided optimum conditions for the DNA polymerase (Supplementary Figure S1). We assume that co-eluted compounds scavenge or mask Mg^2+^ ions, impeding the processivity of the polymerase and leading to increased Cq-values if Mg^2+^ was not supplemented.

For comparison of the DNA extraction kits and calculation of the achieved DNA recovery rates, the Cq-values of the dilution series were plotted against the calculated gene copy numbers in the qPCR reactions along with a 1:10 dilution series of the spiked *E. coli* suspension as the reference.

### 2.4. DNA Extraction Ring-Trial

In a ring-trial of five laboratories (partners A–E), DNA was extracted from a set of biogas fermenter samples with different physicochemical properties following their established adapted DNA isolation routine protocols (1–5 in Figure 1). The extracts were used to study the impact of the DNA extraction protocol on the results of qPCR-based DNA analysis. All five project participants isolated genomic DNA from eight biogas reactor samples (two mesophilic and two thermophilic “hydrolysis” reactors, and two mesophilic and two thermophilic single-stage CSTRs). Four partners (A–D) used DNA extraction protocols involving the FastDNA™ SPIN Kit for Soil (protocols 1–4) due to previous experience that this system combines ease of operation and optimum DNA recovery rates for this type of samples [5], (see also Section 3.1). These protocols mainly differed in applied sample volume, sample treatment and lysis conditions (Table 1). In DNA extraction from the CSTRs, the applied sample volume was limited to 40 or 50 μL (Table 1) due to the previous experience with cattle manure [5], and similar CSTR sludges (not shown) that a higher load can result in co-elution of PCR inhibitors resulting in compromised PCR efficiency or even PCR failure, particularly with undiluted extracts. DNA concentration and quality of the extracts were determined by examination of absorbance at 230, 260, 280 and partially 320 nm, and by the ratios A260/280 and A260/230, as indicated in Supplementary Table S2. Partners A and D used the Quant-iT™ PicoGreen^®^ system (Invitrogen Ltd., Paisley, UK) for DNA quantification and calibration [22].

**Table 1:** DNA extraction protocols for fermenter samples.

Protocol		1	2 *	3 *	4	5
extracted sample volume ^#^	percolate (HR)	2 mL	100 μL	40 μL	2 mL	26 g
	sludge (CSTR)	50 μL	40 μL		50 μL	26 g
pretreatment of samples			60 s Ultra-Turrax, centrifuged, 2× 0.85% KCl-washed		centrifuged, resuspended in ultrapure H_2_O	washed with PBS
cell lysis		2× 20 s @ 5.0 bead beater (MP Biome-dicals, Lysing Matrix E Tubes)	40 s @ 6.0 bead beater (MP Bio-medicals, Lysing Matrix E Tubes)		disruptor (Scientif. industr.); MP Bio-medicals Lysing Matrix E Tubes	CTAB/pronase ε/SDS/65 °C
involvement of a kit		FastDNA Spin kit for Soil				no
DNA-purification		kit components, silica matrix based, no post-purification				chloroform:iso-amylalcohole, isopropanol
elution volume ^#^		100 μL			75 μL	100 μL

* Following the extraction routine described in [5,13] with modifications as indicated; **^#^** all calculations are for 1 mL or 1 g of fresh fermenter sludge or percolate sample.

Extraction procedure 5 (Table 1) was included as a widely applied reference since it followed a CTAB-chloroform:isoamylalcohol routine for DNA isolation: 26 g of sample were mixed with 50 mL of 1 M phosphate buffered saline solution (PBS, 137 mM NaCl, 2.7 mM KCl, 10 mM Na_2_HPO_4_, 1.8 mM KH_2_PO_4_). The mixture was centrifuged at 9000× *g* for 5 min. Pellet resuspension in 50 mL PBS (4 °C), shaking at 400 rpm, centrifugation at 200× *g* for 5 min and supernatant collection followed and was repeated two more times. The collected supernatant was centrifuged at 9000× *g* for 5 min, and the pellet was resuspended in 40 mL PBS and centrifuged again at 5000× *g* for 15 min. The supernatant was discarded. For cell lysis, the pellet was resuspended in CTAB containing DNA extraction buffer (DEP, described previously in Henne *et al.*, 1999) with 5 mg Pronase ε (Serva Electrophoresis GmbH, Heidelberg, Germany) and 2 mg RNase (Qiagen, Germantown, MD, USA) and shaken at 180 rpm and 37 °C for one hour. The suspension was incubated at 65 °C for 2 h after adding 3 mL of 10% SDS solution, while inverting every 15 min. Centrifugation at 3900× *g* for 10 min, filtering through a folded filter (pore size 15–18 μm) and mixing the filtrate 1:1 (v/v) with a 24:1 chloroform/isoamylalcohol (v/v) mixture were followed by centrifugation at 8000× *g* and 4 °C for 5 min. The upper phase was mixed 1:0.7 (v/v) with isopropyl alcohol and incubated at room temperature for one hour. DNA was pelleted by centrifugation at 9000× *g* and 4 °C for 20 min. For DNA purification, NucleoBond AX-G (Macherey-Nagel, Düren, Germany) ion exchange columns and solutions were used in accordance with the manufacturer's protocol. The DNA pellet was resuspended in 2 mL N2-buffer, incubated overnight at 60 °C, and after following the instructions, DNA was eluted with 100 μL TE buffer (10 mM Tris, 1 mM EDTA, pH 8.0) overnight at 4 °C. DNA was quantified using a NanoDrop 2000 Spectrophotometer (Thermo Scientific, Waltham, MA, USA) and was used additionally for DNA qualification and evaluation of contaminants.

Noticeably, DNA preparation routine 5 is typically used to obtain long and unfragmented double-stranded DNA e.g., for whole microbial genome and metagenome analysis. This preparation method must thus confer other qualities than protocols 1–4 and 6 (see Section 2.4) which were developed for representative community composition analysis using a combination of physical cell disruption and chemical lysis, and for sensitive PCR-based quantification of relatively short nucleic acid targets. For this purpose, DNA loss during the extraction procedure and separation of PCR inhibitors are minimized, and the most important goal is to provide the maximum possible recovery rate of PCR-suitable DNA.

The five DNA preparations were sent to four project partners (A–D, Figure 1) who compared DNA quality by laboratory specific qPCR-based quantification of bacterial 16S rDNA, archaeal 16S rDNA and *mcr*A/*mrt*A gene copy numbers of methanogenic *Archaea* (see Section 2.7). 16S rDNA is the target of choice if the diversity of a specified microbial community is the focus, and *mcr*A/*mrt*A is used to specifically determine methanogenic *Archaea*. As a gene encoding subunit A of methyl-CoM-reductase, the key enzyme of methanogenesis, its transcription and translation are a prerequisite for methane production. It is intimately linked with energy production and therefore essential for activity and viability of methane producing *Archaea*.

### 2.5. Preservation Methods for RNA Stability

In a preliminary experiment, samples from the four CSTRs operated by partner B (see Section 2.2) were taken in repetition, and RNA was extracted immediately (see Section 2.6), or after treatment with quaternary ammonium salts as supplied in commercial RNA*later*^®^ (Ambion, Carlsbad, CA, USA) in accordance with the manufacturer’s protocol and after treatment with “homemade” RNA preservative (http://sfg.stanford.edu/RNAbuffer.pdf, http://www.protocol-online.org/prot/Protocols/RNAlater-3999.html; final concentrations: 25 mM sodium citrate, 10 mM EDTA, 70 g ammonium sulfate/100 mL solution, pH 5.2) and storage at −20 °C overnight to simulate a “worst case scenario”. Since commercial RNA*later*^®^ performed slightly better than “homemade” RNA preservative in RT-qPCR evaluation of extracts by partner/analyst B targeting *mcr*A/*mrt*A (see Section 2.7), and almost 60% of *mcr*A/*mrt*A cDNA was quantified relative to the fresh samples (see Section 3.3.1, Figure 6), preservation by commercial RNA*later*^®^ was chosen for sample shipping.

In order to identify possibly even more suitable preservation methods for RNA stabilization in biogas fermenter samples, four methods were tested with digestate from the HR of the two-stage systems (see Section 2.2) operated by partner A (Figure 1); (a) flash freezing in liquid N_2_ and (b) freezing at −80 °C; (c) treatment with RNA*later*^®^; and (d) treatment with formaldehyde. Digestates treated with RNA*later*^®^ stabilization solution (c) were incubated at 4 °C overnight, according to the protocol of the supplier, and then frozen at −80 °C. In method (d), samples were treated with 1.25 mL 1x phosphate buffered solution (PBS) and 3.75 mL of 3.7% formaldehyde. After incubation overnight at 4 °C, the solution was removed subsequently, and samples were washed twice with 1× PBS and stored at −80 °C in a 1× PBS/96% ethanol (1:1, v/v) solution. The frozen samples were sent in dry ice to project partner B for RNA extraction (see Section 2.6).

### 2.6. RNA Extraction and Reverse Transcription

After two months of storage at −80 °C, RNA extraction was carried out (protocol 6 in Figure 1) by partner B with 40 μL fermenter samples that were washed twice using the FastRNA Pro™ Soil–Direct Kit (MP Biomedicals, Santa Ana, CA, USA) with a second washing step, as reported by Munk and coworkers [21]. The TURBO DNA-*free*™ Kit (Ambion, Carlsbad, CA, USA) was used to digest co-extracted DNA in 20 μL eluate in accordance with the manufacturer’s instructions.

Two different reverse transcription (RT) reactions were performed by partner B (Figure 1). The first reaction aimed at transcribing total RNA with random hexamer primers provided in the AffinityScript™ Multiple Temperature Reverse Transcriptase (Agilent Technologies, Santa Clara, CA, USA) kit. Complementary DNA (cDNA) was dedicated to partner A for assays targeting 16S rRNA of *Bacteria* and *Archaea*. The second reaction was specific for *mcr*A*/mrt*A mRNA using reverse primer MeA-i 1435r (Table 1). RT reactions were performed with 5 μL RNA, 200 ng hexamer primers or 600 nM MeA-i 1435r, 2 μL AffinityScript RT buffer (10x), 2 μL DTT (100 mM), 2 μL dNTPs (10 mM) and 1 μL AffinityScript RT filled up with RNase free H_2_O to a final volume of 20 μL. The RT reaction was performed at 42 °C or 45 °C for 60 min for the reactions with random hexamers and *mcr*A*/mrt*A, respectively, and stopped by heat inactivation for 15 min at 70 °C. cDNA was kept on ice for subsequent *mcr*A*/mrt*A qPCR analysis by partner B (see Section 2.7) or was frozen at −20 °C and shipped to partner A on ice for quantification of 16S rRNA transcripts (see Section 2.7).

Protocol 6 was used for the RNA preservation experiment because it was developed for representative community analysis using combined mechanical and biochemical cell disruption and for the sensitive quantification of relatively short RNA fragments by RT-qPCR. This requires minimization of RNA loss during the extraction procedure and should provide a maximum recovery rate of RT-qPCR-suitable RNA. However, preliminary results (see Section 3.3.2) showed that protocol 6 may not fulfill the requirements for metatranscriptome studies since RIN values were unsatisfactory (6.4, 5.4 after rRNA depletion) and because the amount of RNA recovered by the miniprep-procedure from the single-stage fermenters operated by partner B was insufficient. For this reason, a classical acid phenol based RNA extraction routine (protocol 7 in Figure 1) is described here with which the required RNA quality and quantity from biogas fermenter sludge were obtained:

In RNA extraction protocol 7, a full-scale biogas plant (BGP_WF in [23]) was sampled. Fermenter sludge sample was taken by partner E and immediately processed according to a sample volume adapted version of the protocol described by Zoetendal *et al.* [24], followed by application of the RNeasy Midi kit (Qiagen, Hilden, Germany) in accordance with the manufacturer's protocol. In brief, RNA extraction included the following steps (between steps samples were always kept on ice): 4.4 g of fermenter sludge were mixed with 2.5 mL TE buffer (4 °C), applied on a nylon filter (40 μm nylon BD Biosciences, Heidelberg, Germany) and centrifuged at 400× *g* and 4 °C for 2 min. The filtrate was mixed 1 + 1 (v/v) with acid phenol (4 °C), filled into a 15 mL tube with approximately 0.5 g glass beads (0.1 mm) and vortexed for 4 min at highest speed. After centrifugation at 5000× *g* for 5 min, the upper phase was mixed 1:4 (v/v) with RLT buffer (RNeasy Kit, with 2-mercaptoethanol). The following steps were done in accordance with the RNeasy Midi bacteria protocol starting at step 5, with the upper phase/ethanol ratio being 1:2.8 (v/v, without RLT). Finally, RNA was eluted in 50 μL RNase-free water. DNA was digested using the RNase-Free DNase Set (Qiagen, Hilden, Germany) following the manufacturer's instructions. DNA removal was verified by gel electrophoresis. Quality and quantity of the extracted RNA were evaluated using a Prokaryote RNA 6000 Pico chip and the Agilent 2100 Bioanalyzer (Agilent, Santa Clara, CA, USA). For metatranscriptome sequencing, depletion of rRNA from the whole RNA sample [25] was done once using the Ribo-Zero rRNA Removal Kit (Bacteria) (Epicentre, Chicago, IL, USA) and measured again using the Agilent 2100 Bioanalyzer.

### 2.7. Real-Time Quantitative PCR Assays

For evaluation of the DNA extraction ring-trial (Section 2.4) and of the RNA preservation methods (Section 2.5), DNA or cDNA was quantified applying different qPCR-assays. These were targeting bacterial or archaeal 16S rRNA encoding genes and transcripts (measured as cDNA), or the methanogenic archaeal community and its activity by analysis of the functional *mcr*A*/mrt*A genes and transcripts. One Cq value was generally subtracted from RT-qPCR results for cDNA to compensate for the first PCR cycle transforming DNA–RNA hybrids into double-stranded DNA. qPCR reactions were mostly carried out in triplicates.

**Figure 2:**
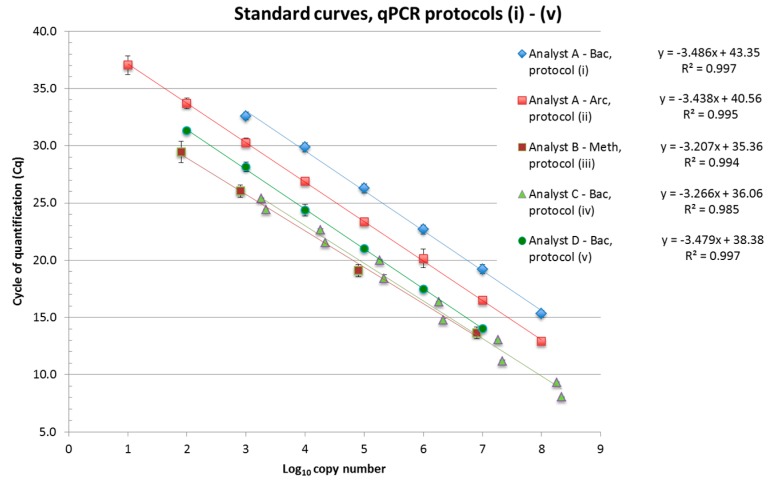
Standard curves applied for the qPCR determination of *Bacteria* (Bac), *Archaea* (Arc) and methanogenic *Archaea* (Meth) in the interlaboratory DNA extraction experiment; partners/analysts A, C and D used plasmid preparations and spectrophotometry to calculate target gene copy numbers, and partner/analyst B applied MPN-qPCR for lysed transformed *E. coli* suspensions.

Partner (and analyst) A applied primers and probes published by Yu and coworkers [26] for the *Bacteria* (Bac, protocol i) and the *Archaea* assays (Arc, protocol ii) with temperature profiles as described in Supplementary Table S1. qPCR reactions were run on a CFX96 Touch™ Real-Time PCR Detection System (BioRad, Sundbyberg, Sweden) using the DyNAmo Flash Probe qPCR Kit (Thermo Scientific, Waltham, MA, USA) in a 20 μL reaction setup. This included 900 nM primers and 200 nM labeled probes (Supplementary Table S1) as final concentration, 10 μL 2× master mix with Mg^2+^ as supplied, 2 μL template, and 4 μL DNase free H_2_O. For the qPCR standards, plasmids containing a target sequence (16S rDNA of *Archaea* [27] and of *Pectobacterium carotovorum* ssp. *carotovorum* DSM 30168 for *Bacteria*) were prepared as described by Klocke *et al.* [27]. The concentration of the linearized plasmid was determined with a NanoDrop^TM^ 3300 fluorospectrometer (Thermo Fisher Scientific Inc., Waltham, MA, USA), and the copy number of 16S rDNA per defined volume was calculated [28]. A tenfold dilution series of these plasmids served for the standard curves (presented as joint projects in Figure 2) with PCR efficiencies of 93.6% and 95.4% for the Bac (i) and Arc (ii) assays, respectively. The single slopes covered a range from −3.4 to −3.6. The Y-intercept ranged between 41.6 and 44.2 for the Bac and 38.4–41.1 for the Arc assays, indicating overestimation particularly of the *Bacteria* target sequences (see also Section 3.2.2). Template (c)DNA was added 10-fold or 100-fold diluted and determined in triplicate for qPCR analyses.

The assay applied by partner/analyst B (protocol iii, Meth) quantifies the methanogenic archaeal community, targeting the functional *mcr*A*/mrt*A genes or their transcripts. Assay design, temperature profile, reactants, preparation of standards and generation of standard curves are described in [13]. Primers MeA-i 1046f/MeA-i 1435r [29] and further details on the assay composition are listed in Supplementary Table S1. Due to their high degeneracy, a higher concentration of 600 nM primers and 6 mM MgCl_2_ were used in qPCR reactions because the DNA polymerase coped best at higher Mg^2+^ concentrations with this type of extracts (see Section 2.3, Supplementary Figure S1). For the standard, an *mcr*A PCR-fragment of a *Methanosarcina* sp. from a CSTR sample was cloned in *E. coli*, and the transformed *E. coli* suspension was propagated and quantified after washing by most probable number (MPN) qPCR analysis of a dilution series of the lysed clone suspension resulting in achieving the target gene concentration in the stock suspension. No signs of qPCR inhibition were seen even for the undiluted stock suspension. More details are found in [13]. The joint project *mcr*A*/mrt*A standard curve obtained with serial clone suspension dilutions (Figure 2) yielded reasonable values for the Y-intercept (Cq 35.4) and the slope (−3.21) resulting in 105% PCR efficiency. Template DNA or cDNA was added undiluted, tenfold and for some extracts 100-fold diluted to reveal PCR inhibition in *mcr*A*/mrt*A qPCR. In –RT reactions (DNase digested RNA extract without Reverse Transcriptase) serving as negative controls, only weak qPCR signals were obtained occasionally. The corresponding amount of residual DNA was negligible, ranging between 0 and 2.8 double-stranded cDNA copies per qPCR assay, and was subtracted from the results to specify the cDNA pool.

Partner C used a SYBR^®^ Green based qPCR system as initially described by Fierer *et al.* [29] for the analysis of *Bacteria* (qPCR protocol iv). For the standard, a PCR product obtained with bacterial 16S rDNA primers AGAGTTTGATYMTGGCTC and CAKAAAGGAGGTGATCC and *Clostridium thermocellum* ATCC 27405 gDNA was cloned into a pJet1.2 blunt end vector. Correct insertion was examined by sequencing. The copy number of inserted nucleotides in the linearized vector was determined by molecular weight calculation [30] and NanoDrop 1000 spectrophotometry. Template (c)DNA was added 10-fold or 100-fold diluted. The joint project standard curve obtained with serial dilutions of the standard displayed reasonable values for the Y-intercept (Cq 36.1) and the slope (−3.27) resulting in 102% PCR efficiency (Figure 2). qPCR assays were performed with the Biorad SsoAdvanced™ Universal SYBR^®^ Green Supermix in a 17 μL setup with 200 nM primers and 2 μL DNA template and run on a CFX96 Touch™ Real-Time PCR Detection System (BioRad, Sundbyberg, Sweden). The temperature profile and more details on the qPCR assay are found in Supplementary Table S1.

Partner (and analyst) D used the same primers and probes for *Bacteria* as partner/analyst A [26]. The LightCycler^®^ 480 (Roche Diagnostics, Basel, Switzerland) with the temperature profile listed in Supplementary Table S1 was used for analysis. Each quantitative real-time PCR assay was carried out in 20 μL of a reaction mixture containing 5 μL of extracted DNA as template, 10 μL of 2× LightCycler^®^ 480 Probes Master (Roche Diagnostics, Basel, Switzerland) with dUTP, 0.1 U Uracil-N-glycosylase (UNG), 500 nM of each primer and 100 nM of the labeled probes (Supplementary Table S1). DNA extracts were diluted 1:100 and 1:1000 and measured in duplicate per dilution. The 16S rDNA plasmid standard was kindly provided by partner/analyst A (see above). The concentration of the linearized plasmid was determined with the fluorescent nucleic acid stain PicoGreen (Quant- iT™ PicoGreen dsDNA Assay Kit, Thermo Fisher Scientific, Waltham, MA, USA) on a SPECTRAmax^®^ GEMINI XS Microplate spectrofluorometer (Molecular Devices, Sunnyvale, CA, USA). After calculation of the copy number of 16S rDNA per defined volume [28], a 10-fold dilution series from 10^7^–10^2^ copies of 16S rDNA per PCR reaction was established and used as the standard. For each qPCR experiment, a plasmid dilution series was prepared and measured in triplicate. For experiments of partner D, Y-intercept and slope of the standard were 38.4 and −3.48, respectively, resulting in 93.8% PCR efficiency (Figure 2). 

## 3. Results and Discussion

### 3.1. Comparison of DNA Extraction Kits and DNA Recovery Rates

**Figure 3:**
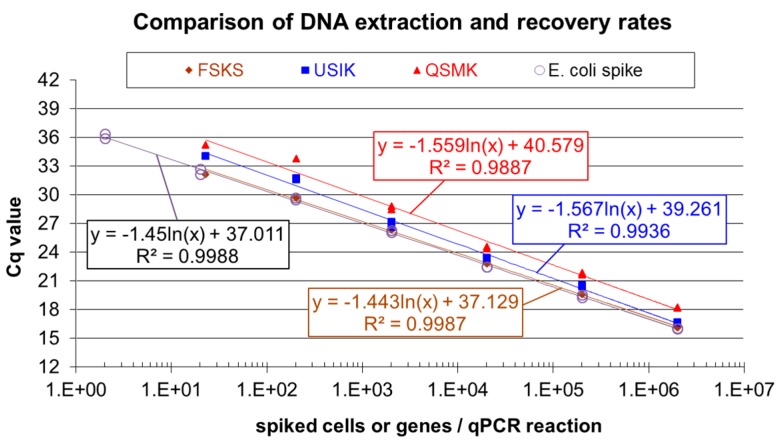
Comparison of DNA extraction efficiencies from treated cattle manure spiked with *Escherichia coli* cells using the FastDNA™ SPIN Kit for Soil (FSKS), the UltraClean^®^ Soil DNA Isolation Kit (USIK) and the QIAamp DNA Stool Mini Kit (QSMK); Cq: cycle of quantification.

In a previous experiment, partner/analyst B spiked cattle manure devoid of *Escherichia coli* DNA with *E. coli* cells and evaluated the efficiencies obtained in parallel DNA extractions with the FastDNA™ SPIN Kit for Soil (FSKS), the UltraClean^®^ Soil DNA Isolation Kit (USIK) and the QIAamp DNA Stool Mini Kit (QSMK) applying *E. coli* specific qPCR. Figure 3 shows the results. Serial extract dilutions yielded similar slopes in *E. coli* *mur*A qPCR (efficiencies 99.30% for the *E. coli* spike, 99.97% for variant FSKS, 89.30% for variant USIK and 89.92% for variant QSMK) with reasonable Y-intercept values (Figure 3). Extraction efficiencies were calculated as means of DNA recovery rates, relating the results for the different extracts obtained at Cq 30 and Cq 20 to the respective whole cell qPCR values for the *E. coli* spike suspension dilutions. Variant FSKS was the most efficient with 88.24%, followed by variant USIK (60.58%) and variant QSMK (37.48%). For comparison, conventional phenol-chloroform-isoamylalcohol extraction of *E. coli* spiked cattle manure did not yield PCR amplifiable DNA (not shown) and was not considered further. Due to the results, DNA extraction procedure FSKS was integrated in the sample processing pipeline of partner/analyst B.

Using the FSKS DNA extraction routine and the Platinum *Taq* polymerase based qPCR assay setup (see Section 2.3), addition of polyvinylpyrrolidone (PVP, 0.85%, 1.7%, 3.4%) and cetyltrimethylammoniumbromide (CTAB, 5%, 10%, 20%, in 1 M NaCl) did not enhance but diminished the extraction efficiency [5], and similarly, addition of T4-gene-32-protein was not found to improve the DNA recovery rate (not shown). These results suggest that such additives as adjuvants might only be helpful in cases of suboptimal nucleic acid extraction or (partially) inhibited PCR but not at an already high recovery rate.

### 3.2. Interlaboratory Comparison of DNA Extraction

Data tracing back to obvious (partially) inhibited PCR reactions, as evidenced e.g., by serial dilution qPCR assays, are consequently excluded from calculations and data processing. However, since identifying and preventing PCR inhibition is extremely important to avoid false negative results, the occurrence of PCR inhibition is discussed in relation to identified or suspected factors.

DNA extracts of all laboratories yielded consistent results in the qPCR assays of the analysts confirming high reproducibility of the analytical procedure. For analytical (qPCR), technical (DNA extraction) and biological (parallel fermenters) replicates, coefficients of variation (CV) were similar (Table 2).

**Table 2:** Coefficients of variation (CV) of analytical, technical and biological replicates. Data represent means of all Bac, Arc and Meth qPCR results over all DNA extracts.

qPCR Assay, Partner/Analyst	Bac (i), A	Arc (ii), A	Meth (iii), B	Bac (iv), C	Bac (v), D
	CV (%)				
analytical	7.56	7.20	7.47	10.10	13.38
technical	20.92	20.80	24.85	n.d.	16.33
biological	28.49	28.70	32.24	25.45	23.27

n.d.: not determined; Bac: *Bacteria*; Arc: *Archaea*; Meth: methanogenic *Archaea*.

#### 3.2.1. Differences between Reactor Types and Operation

**Figure 4:**
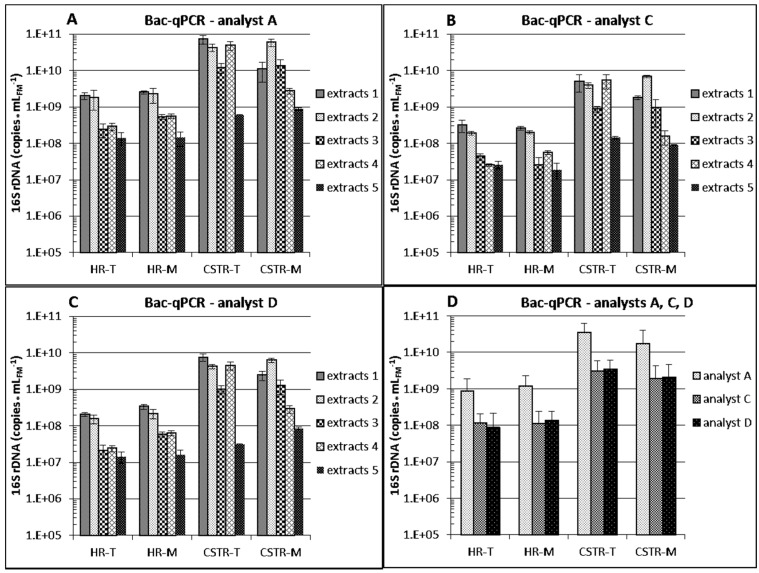
16S rDNA copy numbers for *Bacteria* (Bac) assessed by qPCR and calculated per fresh sample matter as performed by partner/analyst A (**A**); partner/analyst C (**B**) and partner/analyst D (**C**). Means and standard deviations of two biological replicates of four different biogas reactor samples are shown. For each biogas reactor sample, DNA was extracted in different laboratories (partners/analysts A–E) in two technical replicates (1 exception) using their established protocols, and each extract was analyzed in (one exception) two, mostly six and occasionally 10 qPCR replicates. Extracts with the given numbers were obtained with the corresponding DNA extraction protocol. Figure 4D shows the Bac-qPCR data as overall means of extraction protocols 1–5, and the corresponding standard deviations. HR: “hydrolysis” reactor; CSTR: continuously stirred tank reactor; T: thermophilic; M: mesophilic.

**Figure 5:**
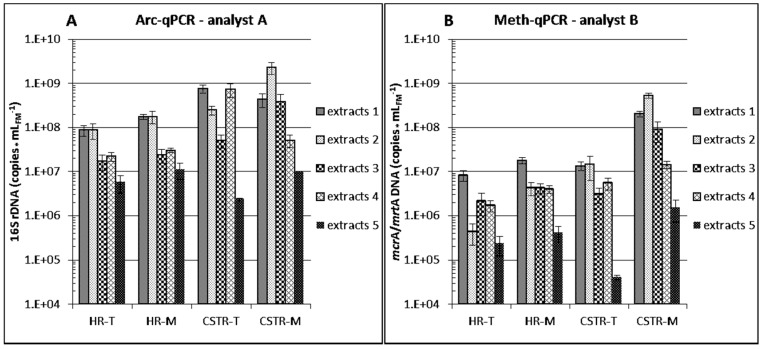
16S rDNA copy numbers for *Archaea* (Arc) and *mcr*A/*mrt*A copy numbers for methanogens assessed by qPCR and calculated per fresh sample matter as performed by partner/analyst A (**A**) and partner/analyst B (**B**). Means and standard deviations of two biological replicates of four different biogas reactor samples are shown. For each biogas reactor sample, DNA was extracted in different laboratories (partners/analysts A–E) in two technical replicates (one exception) using their established protocols, and each extract was analyzed in six, or eight to ten qPCR replicates in case of partner/analyst A or B, respectively. Extracts with the given numbers were obtained with the corresponding DNA extraction protocol. HR: “hydrolysis” reactor; CSTR: continuously stirred tank reactor; T: thermophilic; M: mesophilic.

Total 16S rDNA copy numbers of *Bacteria* (Bac-PCR protocols, Figure 4) and of *Archaea* (Arc-PCR protocol, Figure 5A) differed between reactor types. 16S rDNA copy numbers per milliliter of fermenter material obtained from the CSTR reactors were approximately 10-fold higher than from the HR reactors, independently of the extraction protocol used, no matter if extracts 5 (suboptimal performance, see Section 3.2.3) were considered or not. Only minor differences were detected between 16S rDNA copy numbers at thermophilic or mesophilic conditions in one reactor type, indicating that the tested temperatures (see Section 2.2) did not influence considerably the presence of total *Bacteria* and total *Archaea* in the examined HR and CSTR reactors.

However, the concentration of methanogenic *Archaea* was higher in the mesophilic than in the thermophilic CSTR and HR reactors (Figure 5B, inconsistent data of extracts 5 not considered), although methane productivity rates were almost identical (see Section 2.2). One reason can be a higher richness of methanogens at mesophilic conditions [31,32,33] with lower mean specific activity. In principle, the higher temperature allows for higher activity, and a narrowed spectrum of methanogens can be capable of increased specific metabolic performance [4,34]. Higher specific methanogenic activity at thermophilic conditions can thus explain the observed differences.

The concentration of methanogenic *Archaea* was high in the percolates of the HRs (Figure 5B), and the approximately 10-fold higher concentration of *Archaea* (Figure 5A) supported these data. Since significant amounts of methane were produced not only in the CSTRs but also in the HRs at both temperatures, this confirmed that the methanogens were not only present but displayed high activity also in the HRs.

#### 3.2.2. Differences between Analytical Routines

Figure 4D shows that overall means of bacterial 16S rDNA copy numbers quantified by partners/analysts C and D (protocols iv and v, respectively) for each of the four reactors were similar whereas the overall means determined by partner/analyst A (protocol i) was consistently elevated by one order of magnitude even though the same assay system was used by partners/analysts A and D (Supplementary Table S1). This results from a significant difference in the Y-intercept value of the standard curve of partner/analyst A for *Bacteria* leading to a higher copy number count deduced from the obtained Cq values (Figure 2). The corresponding Y-intercepts of partners/analysts C and D were in a normal range whereas the value of partner/analyst A was unusually high [19]. The high value can be due to DNA degradation or overestimation of the DNA standard template, both of which result in overestimation of calculated copy numbers in samples. No such difference could be observed for archaeal 16S rDNA copy numbers of partner/analyst A (Figure 5A), and the corresponding standard curve Y-intercept value was in the upper normal range (Figure 2). These findings emphasize the necessity of including all necessary data demanded by the MIQE guidelines [9,10,35] to be able to evaluate the comparability of different studies when absolute gene or transcript copy numbers are compared.

A critical issue is also the sensitivity of the assay. For any quantitative assay, the method detection limit (MDL) should be provided. However, the optimum MDL is not achieved with compromised samples, e.g., if DNA is too fragmented or extracts are containing PCR inhibitors [18,36]. PCR inhibition is typically detected in dilution-PCR. Adding e.g., 100-fold diluted DNA template to the reaction mixture will reduce the possibility of PCR inhibition by co-extracted compounds but lower the assay sensitivity by a factor of 100 at the same time. Negative results (below the detection limit) may thus be obtained in case of low target organism abundance in the sample. Such compromised sensitivity relativizes the applicability of the assay. Analyst A used 2 μL of 1:10 or 1:100 diluted DNA template in 20 μL reaction volume (cf. Supplementary Table S1) and observed no PCR inhibition. Analyst B used 1 μL of undiluted, 1:10 and 1:100 of diluted DNA template in 25 μL reaction volume and observed PCR inhibition only with undiluted template for three of four extracts obtained with protocol 4 (by partner D) from the mesophilic CSTR reactors. Analyst C used 2 μL of 1:10 diluted DNA template in 17 μL reaction volume and observed three single inhibited qPCR reactions in total for extracts from the thermophilic CSTR1 obtained with protocols 1, 2 and 5 (by partners A, B and E, respectively). Analyst D used 5 μL of 1:100 and 1:1000 diluted DNA template in 20 μL reaction volume and observed one possibly inhibited PCR reaction with an extract from a thermophilic hydrolysis reactor prepared with protocol 3 (by partner C). Among the tested variants, no clear connection between PCR inhibition and analytical partner could be identified, but the highly viscous CSTR samples obviously presented the greatest challenge for the tested DNA extraction systems.

#### 3.2.3. Differences between DNA Extraction Protocols

Four of the five tested DNA extraction variants (Table 1) involved the FastDNA™ SPIN Kit for soil whereas procedure 5 followed a CTAB-chloroform:isoamylalcohol routine. When mean values over the different analysts and reactor types were calculated per milliliter (or g) fresh sample matter for the five extracts (Table 3), protocol 2 yielded the highest copy numbers followed by protocols 1, 4 or 3, and, finally, protocol 5 in Bac, Arc- and Meth-qPCR.

**Table 3:** Performance of five DNA extracts in different qPCR assays, mean for all analysts and fermenters.

DNA Extracts Obtained with	Bac-qPCR (i, iv, v)	Arc-qPCR (ii)	Meth-qPCR (iii)
	Mean over Analysts and Fermenters (copies · mL_FM_^−1^)		
protocol 1	8.79 × 10^9^	3.64 × 10^8^	6.09 × 10^7^
protocol 2	1.07 × 10^10^	6.99 × 10^8^	1,38 × 10^8^
protocol 3	2.54 × 10^9^	1.18 × 10^8^	2.47 × 10^7^
protocol 4	5.26 × 10^9^	2.08 × 10^8^	6.38 × 10^6^
protocol 5	3.52 × 10^8^	7.17 × 10^6^	5.42 × 10^5^

Bac: *Bacteria*; Arc: *Archaea*; Meth: methanogenic *Archaea*; FM: fresh matter.

The highest microbial DNA yield was thus obtained for all analysts and fermenters with extraction protocol 2 but it appeared to be best suited for qPCR analysis only with the extracts from the more viscous CSTR samples. DNA extraction protocols 1, 3, 4 and 5 yielded only 43%, 19%, 19% and 1% in means of the copies obtained with protocol 2 for CSTR sludge samples, respectively. Protocol 2 provided about 90% DNA recovery in previous spiking experiments with cattle manure (see Section 3.1) and can be modified to warrant similar DNA recovery rates for recalcitrant organisms or mixed communities in a ramping approach with pooling of extracted sub-fractions [37,38]. However, DNA extracts 1 (obtained with protocol 1, provided by partner A) yielded the highest copy numbers for percolate samples from HR reactors by all four analysts (Figure 4A–C and Figure 5). DNA extraction protocols 2, 3, 4 and 5 yielded only 69%, 17%, 18% and 6% in means of the copies obtained with protocol 1 for HR percolates, respectively. One of the reasons can be that it was possible to apply a higher sample volume (2 mL, Table 1, except for protocol 5, see below) of the more liquid HR percolates without risking PCR inhibition. However, 2 mL percolate was also applied in protocol 4 (Table 1) which yielded only 18% of the copies obtained by protocol 1. This might be due to suboptimal cell disruption obtained with the different disruptor device (Table 1). Another reason for the minor performance of protocol 2 as compared to protocol 1 with the HR samples is that analyst B determined relatively low concentrations of methanogens for all four protocol 2 extracts from the two thermophilic HRs (Figure 5B). Since these extracts yielded the highest numbers of archaeal 16S rDNA (Figure 5A) and were among the best in the analysis of *Bacteria* (Figure 4), a problem leading to underestimation may have occurred during the analytical process of analyst B but probably not at the extraction level. It can be recorded that applying a higher volume of HR percolates in extraction protocols 1–4 was of advantage by improving the method detection limit and statistics, since these percolates were more liquid and contained less organic matter and microbial biomass than the more viscous CSTR samples for which protocol 2 provided the best trade-off between optimum qPCR assay sensitivity (method detection limit) and risk of PCR inhibition.

Protocol 5 yielded the lowest copy numbers in all analyses (Figure 4A–C and Figure 5, Table 3). Because of insufficient DNA yields (see also Supplementary Figure S2), partner E sent only one of four planned extracts from the thermophilic CSTRs to the partners. Protocol 5 was thus found to be the least suited for DNA extraction from biogas reactor samples if high DNA extraction efficiency for sensitive downstream qPCR analysis is the goal. However, it must be recalled that this method has been developed to obtain relatively long, unfragmented DNA double-strands, suitable, e.g., for whole microbial genome and metagenome analysis (see Section 2.4). Whether protocols 1–4 are suitable for these applications is currently being tested more exhaustively and is discussed in Section 3.3.2.

DNA concentration in extracts and suitability of extracted DNA for downstream applications such as PCR are traditionally quantified and qualified by spectrophotometric analysis of absorbance at 260 nm and ratios A260/280, A260/230 [12]. Although the respective benchmarks undoubtedly qualify nucleic acid extracts as (almost) pure, some application such as qPCR with specialized novel enzymes obviously do not necessarily require reaching all of these high standards, particularly an A260/230 ratio of >1.8, and the considerable labor investment to reach these levels. However, this depends on the extraction system used: high A230 values are obtained in the presence of co-eluted GITC but do not compromise PCR [13] in contrast to the presence of PCR inhibitors such as phenol. One of the tasks of this work was thus to substantiate this finding in the interlaboratory ring-trial and to figure out important characteristics for PCR-suitable DNA extracts.

As expected, all of the DNA extracts, except for those provided by partner E (extracts/protocol 5) which did not use the FastDNA Spin Kit for Soil with GITC but a CTAB/pronase ε/SDS system instead (Table 1), showed consistently very low A260/230 ratios with a maximum of 0.146, whereas the extracts provided by partner E yielded considerably higher A260/230 ratios, but only one extract exceeded 1.8 (Supplementary Table S2). About half of the extracts showed A260/280 ratios between 1.7 and 2.0, several were lower, probably due to suboptimal exclusion of proteins, and some were higher, even reaching values above 3 (Supplementary Table S2). However, there was no obvious evidence that A260/230 ratios lower than 1.8 or with A260/280 ratios below 1.7 or above 2.0 were responsible for qPCR inhibition: clear qPCR inhibition was only observed by analyst/partner B for four undiluted extracts (CSTR1-M2 by partner A; CSTR1-M1, CSTR2-M1, CSTR2-M2 by partner D) but many other extracts with worse A260/280 and A260/230 ratios did not inhibit the qPCRs. None of the A320 values were above 0.05, the level at which co-eluted humic compounds appeared to (partially) inhibit qPCR [13]. The extract with the highest value (0.044, Supplementary Table S2) did not compromise qPCR. There was thus no indication that extracts as prepared by one of the proposed protocols and missing the mentioned benchmarks of sample purity will fail or are prone to fail in qPCR. However, A260/280 values >2.5 cannot simply be explained by the presence of RNA and pH shifts. Most abnormally high A260/280 values and most inhibited qPCR reactions were observed for the CSTR extracts (Supplementary Table S2). The inhibitor may particularly be produced in mesophilic anaerobic digestion of grass silage and have an absorbance maximum at 260 nm. It remains to be noted here that even very low A260/230 ratios originating from co-elution of GITC are no indicator of qPCR inhibition.

The results of DNA quantification were in general agreement with those of the qPCR approaches, both confirmed higher DNA concentrations in the CSTR sludges than in the HR percolates (Supplementary Figures S2 and S3). However, DNA quantification yielded partially variable results. For several extracts, differences up to a factor of ca. 10 between the results obtained by the different analysts were not rare (Supplementary Figure S2). Strikingly, all DNA concentrations determined for the CSTR extracts 1–4 by partners/analysts B, C and E which applied the extinction coefficient for double-stranded DNA were considerably higher than those determined by partners/analysts A and D which used the PicoGreen^®^ system (Supplementary Figure S2). This was not the case for both, CSTR and HR extracts 5 from partner E, who applied a different extraction system and did not use GITC. Differences between the DNA quantification methods were less evident for the HR extracts, probably due to the low recovered DNA concentrations (Supplementary Figure S2). Besides strong absorption at 230 nm, GITC shows weak absorbance at 260 nm. We assume therefore that DNA concentrations calculated with the extinction coefficient (at 260 nm) were slightly biased for extracts obtained with the FastDNA™ Spin Kit for Soil. However, this cannot explain the considerable overestimation observed with the extinction coefficient for the extracts that were derived from the CSTRs. We assume that an impurity, possibly the inhibitor mentioned above, particularly present in the CSTR extracts operated with grass silage was responsible for the high A260 values.

In conclusion, DNA quantification with the PicoGreen^®^ system appeared to produce more reliable results. Considering only these data (Supplementary Figure S3), DNA concentrations calculated per milliliter or gram of fermenter percolate or sludge were by far higher for the CSTRs. For both, HRs and CSTRs, they were lowest in extract 5 provided by partner E, as discussed above, and highest at almost the same level in extracts 1 and 2 (by partners A and B). Extracts 3 and 4 (by partners C and D) were intermediary. For extract 4, a reason may be that a disruptor was used (Table 1) with which microbial cells may not have been broken up as efficiently as with the bead beater. The low applied percolate volume of only 40 μL (Table 1) resulting in signals partly below the detection limit may explain the relatively low values for HR, but since no such reason could be identified for the CSTR extract 3, we assume that a systemic problem in the extraction procedure of partner C may have occurred.

### 3.3. RNA Experiments

#### 3.3.1. RNA Preservation

**Figure 6:**
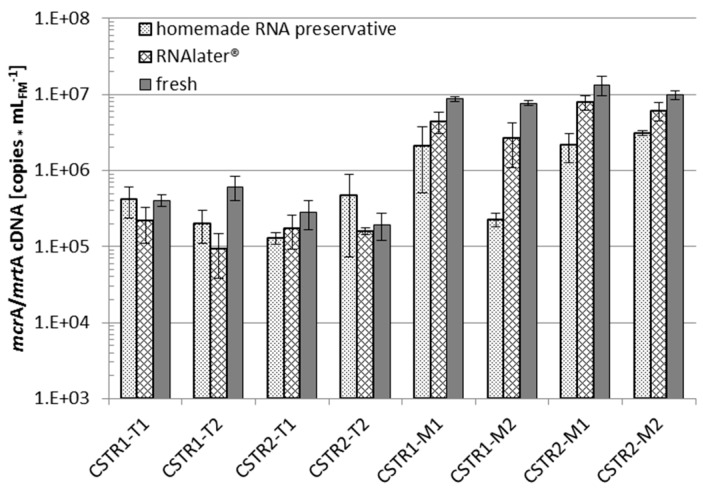
RT-qPCR analysis of *mcr*A/*mrt*A mRNA (analyst B) extracted from two mesophilic (M1,2) and thermophilic (T1,2) biogas CSTR sludge samples in parallel (CSTR1,2). RNA was preserved with commercial RNA*later*^®^ and “homemade” RNA preservative or immediately extracted (fresh). All qPCR analyses were accomplished in triplicate.

RNA preservation was initially tested with commercial RNA*later*^®^ and “homemade” RNA preservative in relation to immediate RNA extraction for samples from the four CSTRs operated by partner B (see Section 2.2). The results are shown in Figure 6.

In comparing the medians of the eight extracts relative to the immediate extraction (fresh, control), preservation by RNA*later*^®^ yielded 57% of the control *mcr*A/*mrt*A cDNA with a SD of 19%, whereas preservation by “homemade” RNA preservative produced only 33% of the control *mcr*A/*mrt*A cDNA with a high SD of 74%. The RNA preservation efficiency with nearly 60% as compared to direct sample processing was considered satisfactory. Due to these results, further evaluations were carried out with commercial RNA*later*^®^. The comparison with flash freezing with N_2_ as the widely applied reference method was of particular interest.

**Figure 7:**
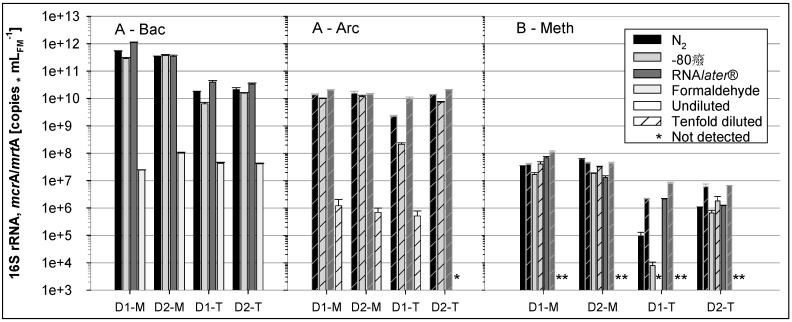
RT-qPCR analysis (analyst A) of bacterial (Bac) and archaeal (Arc) 16S rRNA and of *mcr*A/*mrt*A mRNA (analyst B, Meth) to evaluate the most efficient preservation method for RNA stability: digestates (D) of two (1, 2) mesophilic (M) and thermophilic (T) biogas fermentations (“hydrolysis” stages of partner A) were preserved by flash freezing in liquid N_2_, at −80 °C, treatment with RNA*later*^®^ or formaldehyde. All qPCR analyses were accomplished in triplicate.

The appropriate method for stabilization of RNA was examined for digestates derived from a HR of the two-stage systems operated by partner A by comparing four different preservation methods, formaldehyde treatment, RNA*later*^®^, flash freezing in liquid N_2_ and freezing at −80 °C (see Section 2.5). Figure 7 shows the results of the corresponding RT-qPCR analyses performed by partners and analysts A and B for the RNA extracts prepared by partner B (Figure 1), as described in Section 2.6.

The formaldehyde treatment led to the lowest detection of RNA copy numbers of all preservation methods, and some RT-qPCR signals were even below the corresponding detection limits. The other preservation methods resulted in much higher and mostly similar RNA copy numbers between the variants (Figure 7). Among the three high valued preservation methods, slightly lower RNA copy numbers were obtained for samples that were frozen at −80 °C while RNA*later*^®^ treated samples yielded comparable or higher copy numbers than flash freezing with liquid N_2_ (Figure 7). This is also indicated by Cq factors which were calculated in relation to liquid N_2_ fixation as the reference. Taking into account the data of all RT-qPCR assays, Cq factor medians were 1.5 for RNA*later*^®^ and 0.5 for freezing at −80 °C.

Previous reports on RNA extraction using different sample preservation methods are contradictory: Wang and coworkers [39] obtained equivalent or better RNA quality of cervical tissues preserved by flash freezing with N_2_ than with RNA*later*^®^-preserved tissues. Riesgo and coworkers [40] similarly found for sponge samples that freezing in liquid N_2_ outperformed RNA*later*^®^ in quality and quantity and showed no RNA loss during two months of storage. However, RNA*later*^®^ was favored as a preservation method in studies analyzing tissues, yeast or prokaryotic cultures [15,16,41].

In contrast, there are only very few studies dealing with preservation of RNA from environmental samples. Rissanen and coworkers [17] recommend a phenol-chloroform solution as preservation method for environmental sediment samples containing humic acids. They found that RNA*later^®^* preservation decreased nucleic acid yields drastically and slightly biased community analysis towards certain microbial types. The authors argued that the high content of humic compounds could precipitate nucleic acids by ammonium salts contained in RNA*later^®^*. However, in spite of the fact that the digestates investigated in the current study did contain humic acids, RNA recovery from RNA*later^®^* preservation was at least equivalent with recovery from preservation by liquid N_2_. Moreover, although physical, chemical and structural differences in the mesophilic and thermophilic reactor samples were expected, no major difference between preservation by liquid N_2_ and RNA*later^®^* was observed. Thus, RNA*later^®^* appears to be useful to preserve samples e.g., from agricultural biogas plants where laboratory sample processing chains for RNA are missing.

The bacterial 16S RNA cDNA/DNA ratios for the RNA*later^®^* preservation method indicated very low transcriptional activity, ranging between 0.45 and 1.47 for mesophilic samples, and, in particular, for the thermophilic digestate samples, between 0.06 and 0.1. Lebuhn *et al.* [34] reported higher cDNA/DNA ratios for *Bacteria*, about 10 for mesophilic and about 5 for thermophilic flow-through hydrolytic/acidogenic CSTRs operated with a straw/hay mixture, in spite of the much shorter retention time and lower VS degradation efficiency in the CSTRs and the lower energy content in the straw/hay mixture. Major RNA degradation can be ruled out because almost 60% of the RNA was recovered after preservation by RNA*later^®^* relative to immediate RNA extraction from fresh samples. The lower transcriptional activity can originate from the particular conditions in the “hydrolysis” stages of partner A. Taken together, the results support the efficacy of the highly valued RNA preservation methods.

However, reliable quantitative assessment of RNA species is a challenge in its current state, and results must remain uncertain because solid data on the (m)RNA recovery rate (see below) and RT efficiency are difficult to obtain [42,43]. However, RT efficiency data are an integral part in the assessment of the RT-qPCR efficiency and both data are essential to determine the method detection limit. Quantitative determination of RNA species is not reliable without solid assessment of the RT-qPCR efficiency. It is pointed out that if relative comparisons are intended and one methodology is used in one single experiment, data on the RT-qPCR efficiency are welcome but not absolutely necessary. Comparison of different experiments, however, especially if performed in different laboratories, is critical. If the RT efficiency is not known and absolute quantification of RNA molecules is based on the possibly erroneous assumption that all (100%) of the targeted RNA molecules in the extract are transcribed to cDNA, respective values are speculative and most probably underestimated. Using a pre-quantified enterovirus RNA spike in similar approaches as described in Section 2.3 with the FastRNA^®^ Pro Soil-Direct Kit (see Section 2.6), RNA recovery rates of 70% [4], 30% [21] and 20% [44] were reported, and other RNA extraction systems (RNeasy^®^ Plant Mini Kit (QIAGEN), Strep Thermo-Fast^®^ Plates (ABgene), RNaid^®^ Plus Kit with SPIN™ (Bio 101), QIAamp^®^ MinElute™ Virus Spin Kit (QIAGEN) and QIAamp^®^ Viral RNA Mini Kit (QIAGEN)) yielded only up to 5% RNA recovery [44]. This illustrates the current difficulty of absolute RNA quantification. The scientific community is encouraged to invest more effort in this field.

#### 3.3.2. RNA Qualification and Quantification for Metatranscriptome Sequencing

Metatranscriptome sequencing allows the taxonomic and functional analysis of metabolically active microbial communities on transcript level [45,46,47]. For this purpose, environmental samples are taken and total RNA is extracted. DNA removal, rRNA depletion and cDNA synthesis follow prior to sequencing [45,47,48,49]. Complete DNA removal is a prerequisite because left-over-DNA would be co-sequenced and falsify the sequencing results.

In contrast to RNA extraction protocol 6 aiming at maximizing RNA recovery to provide maximum RT-qPCR sensitivity and to perform representative community analysis, RNA extraction protocol 7 has been designed to extract longer and maximum quality RNA strands, for (meta)transcriptome analysis predominantly consisting of mRNA. Consequently, some RNA loss during the extraction procedure is taken into account. Alternative systems targeting poly-A tails are excluded since a large fraction of prokaryotic mRNA is devoid of poly-A tails.

rRNA removal is essential for metatranscriptome sequencing if considerably deeper sequencing and extensive data filtering is not considered. Messenger RNA (mRNA) in prokaryotic cells only has a share of 1%–20% of the total RNA, while the rest is mostly comprised of rRNA, tRNA and small RNAs. Due to its low percentage, specific depletion particularly of rRNA is important to increase the number of mRNA molecules that harbor the metabolic information of the microbial communities up to one order of magnitude [47,48,50]. The two most frequently used depletion approaches are either based on the enzymatic digestion of rRNAs or on hybridization of these. The enzymatic approach has its drawback in the depletion of degraded mRNAs because this can cause transcriptomic information loss. By contrast, the approach based on hybridization is reported to produce better results and to be more suitable for quantitative analysis [46,47].

**Figure 8:**
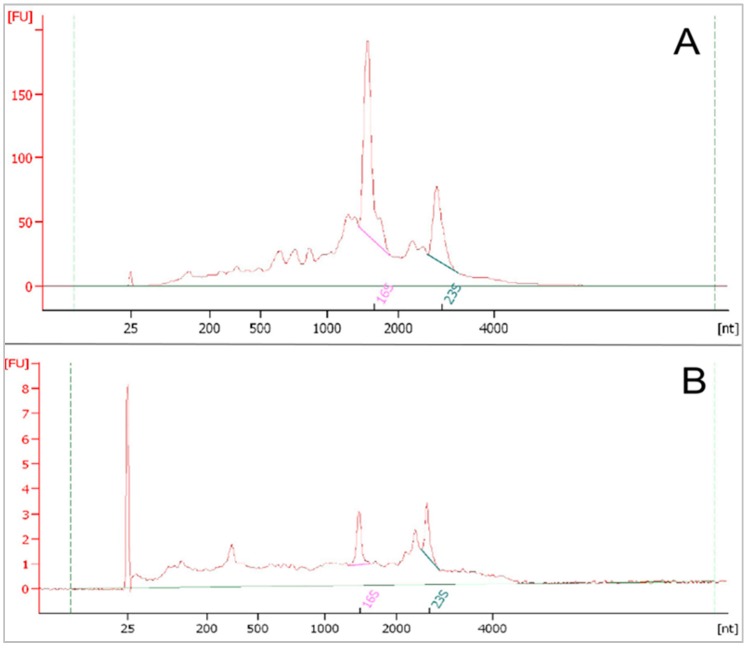
Electropherograms of whole-community RNA extracted from biogas fermenter sludge after DNase digestion. Whole-RNA before (**A**) and after (**B**) rRNA depletion using the Ribo-Zero rRNA Removal Kit (Bacteria) (Epicentre). Fluorescence units (FU) are positively correlated with the RNA concentration. nt: nucleotides.

For this purpose, hybridization based rRNA depletion using the Ribo-Zero rRNA Removal Kit (Bacteria) (Epicentre, Chicago, IL, USA) was chosen (see Section 2.6). Quantity and quality control of the RNA samples was done prior to and after rRNA depletion using the Agilent 2100 Bioanalyzer, resulting in electropherograms as shown in Figure 8. Figure 8A depicts an electropherogram of total RNA extracted from a biogas fermenter and Figure 8B the rRNA-depleted RNA. 23S rRNA has a peak at about 2900 nt and 16S rRNA at about 1540 nt, while mRNAs are typically shorter. The intensity of fluorescence (fluorescence units, FU) is positively correlated with the RNA concentration. For 16S rRNA, the intensity was approximately 67-fold higher prior to depletion than thereafter (approximately 200 FU compared to 3), while for the 23S rRNA fragments, the FU value dropped from approximately 100 to 3. This shows that the depletion was successful for this sample which was prepared following protocol 7.

For metatranscriptome sequencing using whole-community RNA, the RNA integrity number (RIN-value) is important. It indicates the integrity of the RNA and ranges from 1 (completely degraded) to 10 (high integrity, intact) [14]. A challenge is that the RIN value should not be lower than 7 even after rRNA depletion for metatranscriptome sequencing because library preparation and sequencing can be difficult in the presence of considerable amounts of short RNA fragments [14,48].

In contrast, 16S and 23S rRNA peaks were hardly visible in RNA preparations following protocol 6. The RIN value was 6.4 before (Figure 8A) and 5.4 after rRNA depletion (Figure 8B), and RNA yield was not sufficient for metatranscriptome analysis (not shown). If RNA fragmentation or other factors were reasons for the absence of rRNA peaks, precluding rRNA depletion with the given system (see Section 2.6), is a matter of current research. The small sample volume used (see Section 2.6) can explain the low RNA yield. Anyway, protocol 6 cannot be recommended for RNA extraction in the current form with rRNA depletion if metatranscriptome analysis is the aim. As noted by Pascault *et al.* [48], a convenient solution may be, considering the steadily falling sequencing price, to increase the sequencing depth and separate rRNA by bioinformatic tools instead of removing rRNA by technical means.

## 4. Conclusions

An interlaboratory comparison of nucleic acid extraction, RNA preservation and quantitative Real-Time PCR (qPCR) based assays was conducted. Extracts compared in the ring-trial were derived from differently operated biogas fermenters digesting grass silage as difficult matrices. A kit based DNA extraction system with modifications such as upstream sample washing showed excellent DNA recovery rates. In order to optimize the trade-off between PCR suitability and DNA loss during multiple purification steps, less material could be processed with highly viscous fermenter sludge samples, but no post-purification was necessary. qPCR inhibition was observed only in very few cases in dilution assays as an exception. The system produced qPCR-suitable extracts and highly reproducible qPCR results in the ring-trial between five institutional partners, whereas a traditional chloroform-CTAB based protocol performed considerably worse in qPCR analyses of *Bacteria*, *Archaea* and methanogens. Classical absorption coefficients were of limited use to predict qPCR-suitability of nucleic acid extracts from highly viscous biogas fermenter sludges. Particularly, the ratio A260/230 was of no significance when GITC was present in the extracts. As compared to quantification of total DNA with the PicoGreen^®^ system, using the extinction coefficient appeared to generate overestimated results particularly for the CSTR extracts obtained with the kit system, possibly due to interference of a co-extracted impurity. Quantitative data on total DNA contents in extracts from highly viscous fermenter sludges must thus be regarded with precaution, particularly if spectrophotometry is used.

Besides optimal primer design, special attention must be paid to the generation of a reliable standard with a plausible slope and an appropriate Y-intercept value. If this is not the case, considerable over- or underestimation in absolute quantification is the consequence. Respective data for the standard should be provided along with data for the extraction efficiency of the nucleic acid extraction system used.

Several methods were tested in an experiment to evaluate RNA preservation methods. Sample storage in RNA*later*^®^ was at least as efficient as flash freezing and storage in liquid N_2_. As compared to immediate processing of fresh samples, RNA loss of about 40% was observed if samples were stored in RNA*later*^®^. Although there is potential to optimize the system, RNA preservation with RNA*later*^®^ can thus be recommended for field applications where sample shipping is an issue.

Considerable variation and poor reproducibility were observed for the tested total RNA extraction systems with the given fermenter sludge samples. It is not clear if the deficiencies are due to suboptimal extraction efficiency, compromised reverse transcription (RT) or both. Although plausible RT-qPCR results could be generated for distinct experiments, RNA yield and quality was insufficient after rRNA depletion for metatranscriptome analysis. The scientific community is encouraged to invest more effort in this field.

## Supplementary Files

Supplementary File 1
