# Supplementary material for: DNA and RNA Extraction and Quantitative Real-Time PCR-Based Assays for Biogas Biocenoses in an Interlaboratory Comparison
Source: Bioengineering (Basel). 2016 Jan 13;3(1):7. doi: 10.3390/bioengineering3010007 (PMC5597165; doi:10.3390/bioengineering3010007)
Supplement: Supplementary File 1 [file bioengineering-03-00007-s001.pdf]

Supplementary Materials: DNA and RNA Extraction and Quantitative Real-Time PCR-Based Assays for Biogas Biocenoses in an Interlaboratory Comparison

Michael Lebuhn [1,*], Jaqueline Derenkó [2,†], Antje Rademacher [2,†], Susanne Helbig [3,†], Bernhard Munk [1,†], Alexander Pechtl [4,†], Yvonne Stolze [5,†], Steffen Prowe [3], Wolfgang H. Schwarz [4], Andreas Schlüter [5], Wolfgang Liebl [4] and Michael Klocke [2]

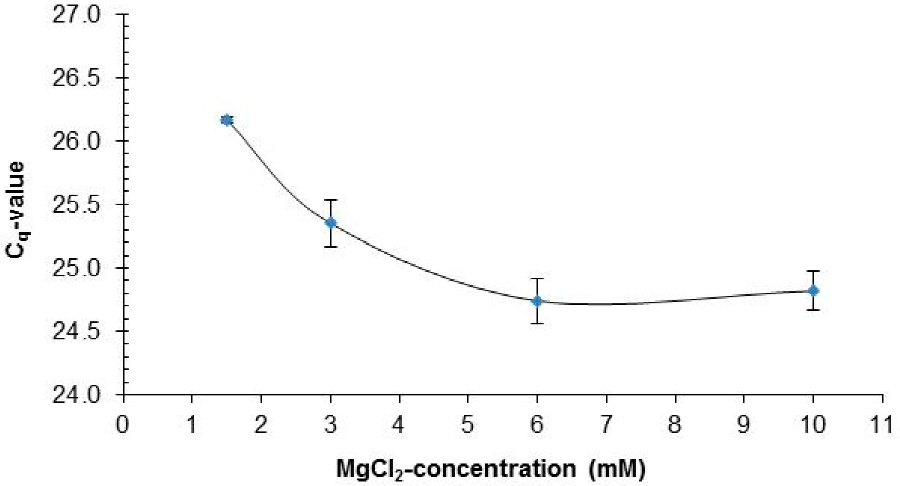

Figure S1. Effect of different $MgCl_2$ concentrations on the qPCR efficacy. DNA from cattle manure spiked with *E. coli* was extracted using system FSKS and quantified using the *mur*A system (see Section 2.3).

Table S1. qPCR assays used in the interlaboratory comparison.

**Partner/Analyst**		**A**	**B**	**C**	**D**
DNA quantification		NanoDrop 1000 + PicoGreen	IMPLEN NanoPhotometer™	NanoDrop 1000	NanoDrop 1000 + Picogreen
qPCR reagents		Thermo Scientific DyNAmo Flash Probe qPCR Kit; Mg^{2+} as supplied by manufacturer;	Self-made master-mix containing 2xEvaGreen, 200 µM dNTPs and 0.75 U Platinum Taq-Polymerase; Mg^{2+} 6 mM in qPCR assays (Lebuhn *et al.*, 2003; Munk *et al.*, 2010);	Biorad SsoAdvanced™ Universal SYBR® Green Supermix; Mg^{2+} as supplied by manufacturer;	LC480 Roche Probes Master; Mg^{2+} 3.2 mM in qPCR assays;
Reaction volume		18 µL master-mix + 2 µL extract	24 µL master-mix + 1 µL extract	15 µL master-mix + 2 µL extract	15 µL master-mix + 5 µL extract
Primers and probes	Bac fw	Bac Fw (ACT CCT ACG GGA GGCAG), [1]		Eub 338 (ACTCCTACGGGAGGCAG), [3]	Bac Fw (ACTCCTACGGGAGGCAG), [1]
	Bac probe	Bac Probe (6-FAM-TGCCA GCAGC CGCGG TAATA C-TAMRA), [1]			Bac Probe (6-FAM-TGCCA GCAGC CGCGG TAATA C-TAMRA), [1]
	Bac rev	Bac-rev (GAC TAC CAG GGT ATC TAA TCC), [1]		Eub 518 (ATTACCGCGGCTGCTGG), [3]	Bac-rev (GACTA CCAGG GTATC TAATC C), [1]
	Arc fw	Arc-fw (ATTAGA TACCCS BGTAGT CC), [1]			
	Arc Probe	Arc-Probe (6-FAM-AGGAA TTGGC GGGGG AGCAC-TAMRA), [1]			
	Arc rev	Arc-rev (GCCATG CACCWC CTCT), [1]			
	mcrA/mrtA fw		MeA-i 1046f (TAYATGWSIGGHGGIGTIGGI TTYAC), [2]		
	mcrA/mrtA rev		MeA-i 1435r (TGRTCYTGIARRTCRWAICCR WAGAAICC), [2]		
qPCR platform		CFX96 Touch (Biorad)	MX 3005P qPCR System (Agilent)	CFX96 Touch (Biorad)	LightCycler480 (Roche)

Table S1. *Cont.*

**Partner/Analyst**		**A**	**B**	**C**	**D**
Protocols	Bac protocols (i, iv, v)	95 °C, 07:00<br>95 °C, 00:15<br>57 °C, 00:30<br>60 °C, 00:60<br>45x		98 °C, 02:00<br>98 °C, 00:05<br>56 °C, 00:35<br>40x	Pre-Incubation<br>95 °C, 10:00<br>95 °C, 00:10<br>60 °C, 00:30<br>72 °C, 00:01 (acquisition)<br>45x
	Arc protocol (ii)	95 °C, 07:00<br>95 °C, 00:15<br>60 °C, 00:60<br>45x			
	mcrA/mrtA protocol (iii)		95 °C, 03:00<br>95 °C, 00:15<br>60 °C, 01:00<br>82 °C, 00:10 (acquisition)<br>45x		

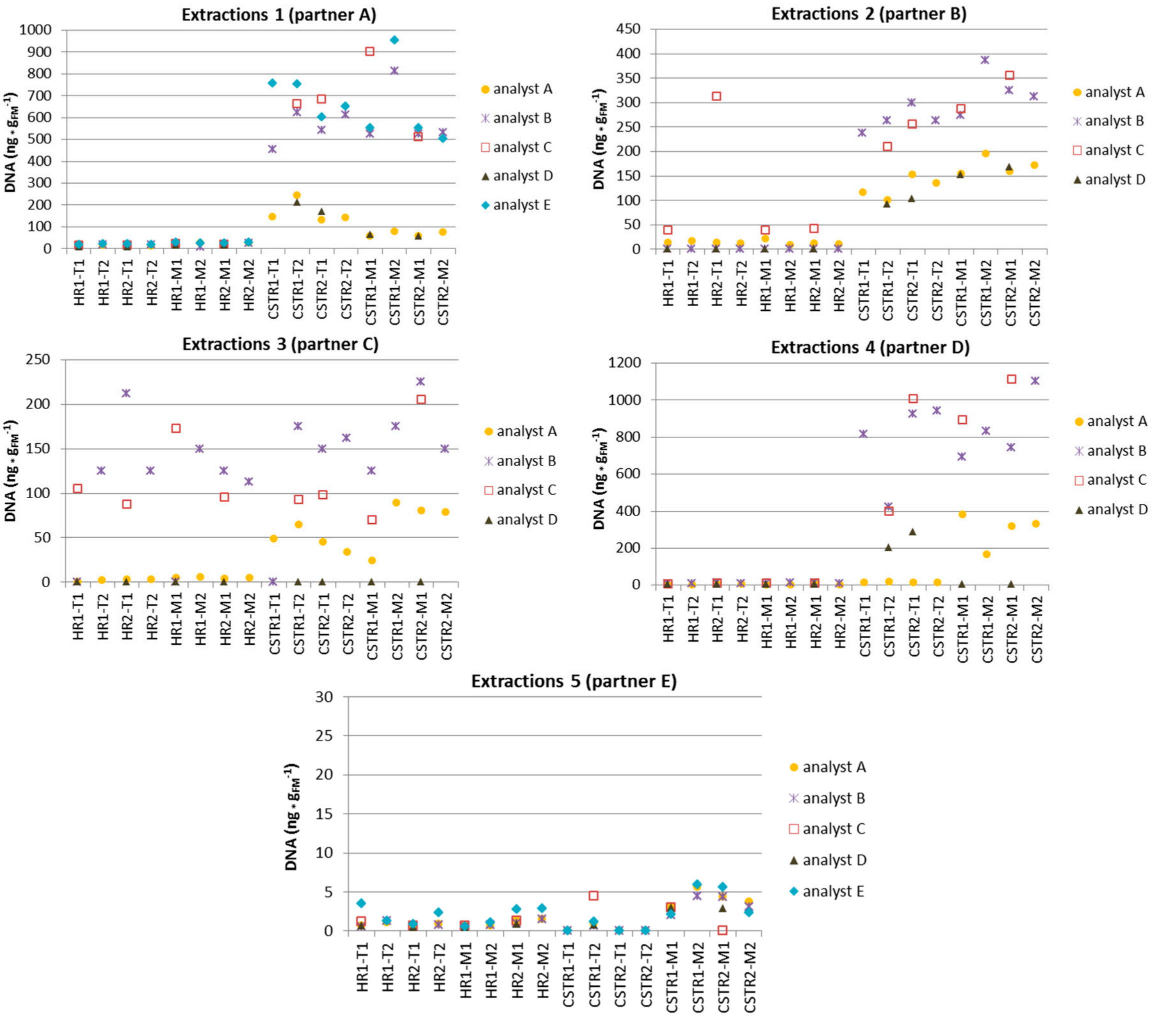

Figure S2. DNA concentrations calculated per mL or g of fresh sample matter of extracts produced by the five partners of the interlaboratory comparison as determined with the extinction coefficient for ds-DNA (analysts B, C and E) or the PicoGreen® system (analysts A, D).

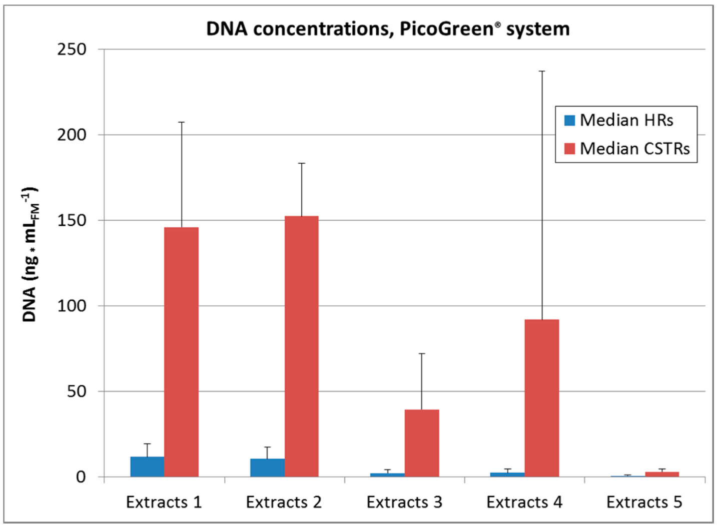

Figure S3. DNA concentrations in the extracts prepared by the partners calculated per mL g of fresh sample matter using only the data from the PicoGreen® system. The medians over the different analysts with the SDs are presented.

Table S2. Absorbance ratios A260/280 and A260/230, and absorbance at 320 nm of extracts produced by the five and analyzed by two partners/analysts of the interlaboratory comparison.

Extracts 1 (Partner A)							Extracts 2 (Partner B)				Extracts 3 (Partner C)			
**Analyst B**	**A260/280**	**A260/230**	**A320**	**Analyst E**	**A260/280**	**A260/230**	**Analyst B**	**A260/280**	**A260/230**	**A320**	**Analyst B**	**A260/280**	**A260/230**	**A320**
HR1-T1	1.767	0.03	0.011	HR1-T1	1.86	0.03	HR1-T1	<d.l.	<d.l.	<d.l.	HR1-T1	2	0.022	0.002
HR1-T2	1.714	0.031	0.014	HR1-T2	1.5	0.04	HR1-T2	<d.l.	<d.l.	<d.l.	HR1-T2	1.667	0.024	0.003
HR2-T1	1.719	0.03	0.014	HR2-T1	1.57	0.04	HR2-T1	<d.l.	<d.l.	<d.l.	HR2-T1	1.7	0.037	0.003
HR2-T2	1.647	0.042	0.014	HR2-T2	1.63	0.05	HR2-T2	<d.l.	<d.l.	<d.l.	HR2-T2	2	0.034	0.004
HR1-M1	1.686	0.042	0.021	HR1-M1	1.54	0.04	HR1-M1	<d.l.	<d.l.	<d.l.	HR1-M1	1.4	0.025	0.003
HR1-M2	*2.75*	0.044	−0.002	HR1-M2	1.42	0.09	HR1-M2	<d.l.	<d.l.	<d.l.	HR1-M2	*2.4*	0.017	0.002
HR2-M1	1.756	0.044	0.015	HR2-M1	1.63	0.05	HR2-M1	<d.l.	<d.l.	<d.l.	HR2-M1	1.667	0.032	0.002
HR2-M2	1.826	0.04	0.024	HR2-M2	1.62	0.04	HR2-M2	<d.l.	<d.l.	<d.l.	HR2-M2	1.8	0.033	0.002
CSTR1-T1	1.957	0.021	0.012	CSTR1-T1	1.67	0.02	CSTR1-T1	1.727	0.052	0.005	CSTR1-T1	0.8	0.016	0.002
CSTR1-T2	1.676	0.032	0.044	CSTR1-T2	1.67	0.03	CSTR1-T2	1.615	0.041	0.004	CSTR1-T2	1.556	0.037	0.003
CSTR2-T1	1.742	0.031	0.015	CSTR2-T1	1.5	0.03	CSTR2-T1	1.6	0.045	0.005	CSTR2-T1	2	0.031	0.003
CSTR2-T2	1.848	0.031	0.015	CSTR2-T2	1.63	0.03	CSTR2-T2	1.5	0.146	0.004	CSTR2-T2	1.625	0.038	0.002
CSTR1-M1	1.793	0.025	0.014	CSTR1-M1	1.38	0.02	CSTR1-M1	1.692	0.14	0.003	CSTR1-M1	1.667	0.033	0.001
CSTR1-M2 *	1.929	0.036	0.028	CSTR1-M2	1.73	0.02	CSTR1-M2	1.632	0.091	0.009	CSTR1-M2	1.75	0.044	0.002
CSTR2-M1	1.926	0.028	0.019	CSTR2-M1	1.38	0.03	CSTR2-M1	1.733	0.058	0.004	CSTR2-M1	1.8	0.1	0.002
CSTR2-M2	1.71	0.028	0.017	CSTR2-M2	1.67	0.02	CSTR2-M2	1.786	0.139	0.005	CSTR2-M2	1.714	0.041	0.001

Extracts 4 (Partner D)				Extracts 5 (Partner E)						
**Analyst B**	**A260/280**	**A260/230**	**A320**	**Analyst B**	**A260/280**	**A260/230**	**A320**	**Analyst E**	**A260/280**	**A260/230**
HR1-T1	1.75	0.019	0.008	HR1-T1	1.765	0.435	0.002	HR1-T1	1.85	1.26
HR1-T2	1.647	0.019	0.008	HR1-T2	1.806	0.699	0.002	HR1-T2	1.76	0.79
HR2-T1	1.833	0.019	0.009	HR2-T1	1.933	0.426	0.001	HR2-T1	1.74	0.66
HR2-T2	1.5	0.061	0.01	HR2-T2	1.905	0.506	0.002	HR2-T2	1.77	1.05
HR1-M1	1.8	0.017	0.007	HR1-M1	1.812	0.403	0.001	HR1-M1	1.78	0.51
HR1-M2	1.684	0.022	0.01	HR1-M2	1.818	0.525	0.001	HR1-M2	1.77	0.67
HR2-M1	1.591	0.031	0.01	HR2-M1	1.759	0.646	0.001	HR2-M1	1.8	1.24
HR2-M2	1.706	0.022	0.008	HR2-M2	1.795	0.814	0.004	HR2-M2	1.82	1.17
CSTR1-T1	*2.077*	0.036	0.016	CSTR1-T1	n.d.	n.d.	n.d.	CSTR1-T1	1.345	0.34
CSTR1-T2	1.909	0.023	0.008	CSTR1-T2	1.783	1.019	0.004	CSTR1-T2	*2.5*	1.12
CSTR2-T1	*2.14*	0.042	0.021	CSTR2-T1	n.d.	n.d.	n.d.	CSTR2-T1	n.d.	n.d.
CSTR2-T2	*2.293*	<d.l.	0.019	CSTR2-T2	n.d.	n.d.	n.d.	CSTR2-T2	n.d.	n.d.
CSTR1-M1*	*2.654*	0.03	0.017	CSTR1-M1	1.763	1.305	0.005	CSTR1-M1	2.04	1.34
CSTR1-M2	*2.306*	0.036	0.025	CSTR1-M2	1.773	1.376	0.005	CSTR1-M2	1.92	1.89
CSTR2-M1*	*2.552*	0.033	0.019	CSTR2-M1	1.775	1.153	0.004	CSTR2-M1	1.93	1.79
CSTR2-M2*	*3.235*	<d.l.	0.022	CSTR2-M2	1.8	0.462	0.002	CSTR2-M2	2.06	1.41

*: qPCR inhibition by undiluted extract; n.d.: not determined/not delivered; <d.l.: below detection limit; red color denotes values below and pink color values above conventional thresholds.

References

1. Yu, Y.; Lee, C.; Kim, J.; Hwang, S. Group-specific primer and probe sets to detect methanogenic communities using quantitative real-time polymerase chain reaction. *Biotechnol. Bioeng.* **2005**, *89*, 670–679.
2. Bauer, C.; Korthals, M.; Gronauer, A.; Lebuhn, M. Methanogens in biogas production from renewable resources—A novel molecular population analysis approach. *Water Sci. Technol.* **2008**, *58*, 1433–1439.
3. Fierer, N.; Jackson, J.; Vilgalys, R.; Jackson, R.B. Assessment of soil microbial community structure by use of taxon-specific quantitative PCR assays. *Appl. Environ. Microbiol.* **2005**, *71*, 4117–4120.

© 2016 by the authors; licensee MDPI, Basel, Switzerland. This article is an open access article distributed under the terms and conditions of the Creative Commons by Attribution (CC-BY) license (http://creativecommons.org/licenses/by/4.0/).
